# Acute Otitis Media in Children—Challenges of Antibiotic Resistance in the Post-Vaccination Era

**DOI:** 10.3390/microorganisms10081598

**Published:** 2022-08-08

**Authors:** Cristina Gavrilovici, Elena-Lia Spoială, Ingrith-Crenguţa Miron, Iuliana Magdalena Stârcea, Codruţa Olimpiada Iliescu Haliţchi, Irina Nicoleta Zetu, Vasile Valeriu Lupu, Carmen Pânzaru

**Affiliations:** 1 Department of Pediatrics, “Grigore T. Popa” University of Medicine and Pharmacy, 16 Universitatii Street, 700115 Iasi, Romania; 2 “St Mary” Emergency Hospital for Children, 700309 Iasi, Romania; 3 Department of Orthodontics and Dento-Facial Orthopedics, “Grigore T. Popa” University of Medicine and Pharmacy, 16 Universitatii Street, 700115 Iasi, Romania; 4 Department of Microbiology, “Grigore T. Popa” University of Medicine and Pharmacy, 16 Universitatii Street, 700115 Iasi, Romania

**Keywords:** acute otitis media, tympanocentesis, *Streptococcus pneumoniae*, antibiotic resistance, children

## Abstract

Acute otitis media (AOM) is a leading cause of antibiotic prescriptions in children worldwide, even in the era of pneumococcal conjugate vaccines. We aimed to assess the bacterial spectrum of AOM in children and to investigate the antimicrobial resistance profile in culture-positive cases. We performed a retrospective, tympanocentesis-based analysis of antimicrobial resistance patterns in children with AOM hospitalized in “St Mary” Emergency Hospital for Children Iasi, Romania, between January 2013 and December 2021. A total of 147 samples have been assessed, 97 (65.98%) of which had positive cultures, with *Streptococcus pneumoniae* and *Haemophilus influenzae* as the most common microorganisms. A worrying proportion, 82.85% (58/70), of the *Streptococcus pneumoniae* strains were multidrug-resistant. The World Health Organization included *Streptococcus pneumoniae* and *Haemophilus influenzae* on the medium priority group due to penicillin non-susceptibility and ampicillin-resistant strains, respectively. Consequently, strategies to address the threats of antimicrobial resistance are needed to reduce the potential negative effects on hospitalization costs.

## 1. Background

Acute otitis media (AOM) represents a leading cause of antibiotic prescriptions in children worldwide [1] and a substantial economic burden on healthcare resources, even in the era of pneumococcal conjugate vaccines [2]. Approximately 80% of all children will experience at least one episode of AOM, with one third of cases with six or more episodes by the age of seven [3].

The microbiological profile and the antibiotic resistance patterns are essential to guide the optimal treatment in AOM in children. While the etiology of AOM is clearly established, with the most prevalent microorganisms represented by *Streptococcus pneumoniae*, *Haemophilus influenzae* and *Staphylococcus aureus*, the antimicrobial resistance data focuses mainly on *Streptococcus pneumoniae*, with limited data on other microorganisms [4]. Worldwide, there are large variations (0–65.8%) regarding the lack of susceptibility of *Streptococcus pneumoniae* to penicillin, due to different pneumococcal conjugate vaccination rates, as well as due to the lack of uniformity in the availability and adherence to AOM guidelines [4]. In order to reduce antibiotic resistance, different guidelines on AOM management encourage a watchful waiting approach rather than immediate antibiotic treatment [5]. However, over-treatment with antibiotics and inaccurate dosing are still reported [6,7]. In a recent study, Frost et al. [8] found that most antibiotic prescriptions for AOM exceeded the duration recommended by the guidelines: 54% of children were treated for at least 10 days, probably due to extrapolations from the streptococcal tonsillitis treatment regimen. The authors underline that the medical decision regarding treatment duration is not always based on the severity of AOM or on the patient’s age [8].

Nowadays, the preferred therapeutic agents for AOM are represented by beta lactams, which act as inhibitors of the bacterial cell wall synthesis [9]. Amoxicillin, the first-line antibiotic for uncomplicated AOM, exerts its bactericidal action by binding to penicillin-binding proteins that inhibit transpeptidation (the cross-linking process in cell wall synthesis) [9]. In case of treatment failure, amoxicillin/clavulanic acid shows excellent response in beta-lactamase-producing *Haemophilus* influenzae and penicillinase-producing anaerobes [10]. This beta-lactamase inhibitor works by binding irreversibly to beta-lactamase, an enzyme which causes resistance to the original beta-lactam ring of amoxicillin [11]. Second-line options include several classes of antibiotics with different mechanisms of action, as follows: cephalosporins (ceftriaxone, cefuroxime)—inhibits bacterial cell wall synthesis similar to amoxicillin; macrolides (azithromycin, clarithromycin, erythromycin) and lincosamides (clindamycin)—inhibit bacterial protein synthesis; fluoroquinolones (levofloxacin)—inhibits deoxyribonucleic acid synthesis; trimethoprim/sulfamethoxazole—inhibits folic acid synthesis [5,12].

The aim of our study was to assess the bacterial spectrum of infection in children (0–18 years old) with AOM in a tertiary hospital in Romania and to investigate the antimicrobial resistance profile.

## 2. Patients and Methods

We performed a retrospective study in children with AOM hospitalized in “St Mary” Emergency Hospital for Children Iasi, Romania, between January 2013 and December 2021. Tympanocentesis was recommended in children with refractory otitis media, in uncomplicated cases with a negative outcome (unsatisfactory response to antimicrobial therapy) and in patients with complications of AOM (e.g., mastoiditis).

The diagnosis was based on the following criteria: acute onset of the symptoms (fever, irritability, otalgia or otorrhea) and middle ear effusion confirmed by otoscopy (bulging or erythema of the tympanic membrane, air-fluid level behind the tympanic membrane, limited or absent mobility of tympanic membrane). Eligible patients were children with a recent onset of AOM symptoms (in the first 48 h) who did not receive any antibiotic treatment or children with a prior diagnosis of AOM who received antibiotic treatment but remained symptomatic (fever or otalgia) for more than 48 h (treatment failure). Children with a previously inserted ventilatory tube or those with spontaneous perforation of the tympanic membrane were excluded from our study.

The samples were assessed for microbiological culture and antibiotic sensitivity testing. The pus obtained by tympanocentesis was used for smears and inoculation on Columbia with 5% sheep blood (Oxoid, Leicestershire, UK) and chocolate agar and Vitox (Oxoid, Thermoscietific, Leicestershire, UK). The smears were stained with methylene blue (for a better appreciation of the polymorphonuclear neutrophils, which, along with the fibrin, is the marker of inflammation) and Gram stain to establish the morpho-tinctorial category of the bacteria. The identification of isolated colonies was carried out with MICROSCAN panels (PC42, HIND) and MALDI-TOF mass spectrometry when applicable. To test the antibiotic sensitivity of *Streptococcus pneumoniae*, we used MSTRP+6 (Microscan, CLSI, Wayne, PA, USA) and STP6F (Sensititre, CLSI, West Sussex, UK) panels for *Haemophilus influenzae*. Additionally, for *Haemophilus influenzae* and *Moraxella catarrhalis*, we used the nitrocefin discs test (Thermoscientific Remel Nitrocefin discs - San Diego, CA, USA) to assess beta-lactamase production. The nitrocefin disk is a disk impregnated with nitrocefin for the detection of β- lactamase by chromogenic procedure. Nitrocefin, a cephalosporin, is the substrate used in this test. β- lactamase hydrolyzes the β- lactam ring of nitrocefin, producing cephalosporanic acid. A distinctive color change is associated with this reaction wherein the pale yellow nitrocefin is converted to a pink end-product upon hydrolysis. The bacteria that do not produce β- lactamase do not alter the pale-yellow color of nitrocefin within the limit of the test (5 min at room temperature). Unfortunately, for *Haemophilus influenzae*, we did not determine the serotype. HIND panels were used to indicate the biotype. *Turicella otitidis* had not been tested with antibiotics. The strains are usually susceptible to beta-lactams, aminoglycosides, glycopeptides, pristinamycin and rifampicin, while susceptibility to fluoroquinolones, macrolides and trimethoprim/sulfamethoxazole is variable. Antimicrobial susceptibility of *Streptococcus pneumoniae* was tested for the following antibiotic classes: penicillins (penicillin), fluoroquinolones (moxifloxacin, levofloxacin), cephalosporins (ceftriaxone, cefotaxime, cefuroxime), macrolides (azithromycin, clarithromycin, erythromycin), trimethoprim-sulpamethoxazole, lincosamides (clindamycin), glycopeptides (vancomycin) and chloramphenicol, whereas the following antibiotics were assessed for sensibility in *Haemophilus influenzae* cases: amoxicillin/clavulanic acid, cefuroxime, ceftriaxone and trimethoprim/sulfamethoxazole. We collected the following health data: demographic features (age and sex), year of diagnosis, the patients’ AOM history, immunization scheme, comorbidities and recent antibiotic treatment, culture and antibiogram results using the electronic medical record provided by our hospital.

The study protocol was reviewed and approved by the Ethical Committees of “Grigore T. Popa” University of Medicine and Pharmacy, Iasi, Romania (No. 12292-22.07.2020) and of “St Maria” Clinical Emergency Hospital for Children, Iasi (No. 34306-05.10.2020).

## 3. Results

### 3.1. Sample Description. Bacterial Spectrum of AOM

A total of 147 samples from children between 2 months to 7 years old (mean age: 19 months old) were assessed, 97 (65.98%) of which had positive cultures as listed above: 70 positive for *Streptococcus pneumoniae*, 17 for *Haemophilus influenzae*, 2 for *Moraxella catarrhalis*, 2 for methicillin-resistant *Staphylococcus aureus* (MRSA), 2 for *Pseudomonas aeruginosa*, 1 for *Escherichia coli*, 1 for *Enterobacter aerogenes*, 1 for *Streptococcus pyogenes*, and 1 for *Turicella otitidis* (Table 1). All the children have been immunized according to our national vaccination scheme in our country, which includes vaccinations against tuberculosis, diphtheria, tetanus, pertussis, poliomyelitis, *Haemopilus influenzae* type b infection, hepatitis B, measles, mumps and rubella. None of the children were immunized against pneumococcal disease, as this vaccine is included in the national vaccination calendar depending on available funds.

### 3.2. Antimicrobial Resistance Profile

We focused on *Streptococcus pneumoniae* and *Haemophilus influenzae* (70/147, and 17/147, respectively), as these strains were isolated most frequently (89.68% of all positive cultures. The penicillin resistant strains of *Streptococcus pneumoniae* accounted for 5.71% (4/70), whereas 10 strains (14.28%) had intermediate susceptibility to penicillin. We report the minimum inhibitory concentrations for *Streptococcus pneumoniae* strains (Table 2). In vitro sensitivity for ceftriaxone, moxifloxacin and vancomycin was reported in all tested strains, whereas only 6/70 strains were susceptible to trimethoprim/sulfamethoxazole, and 9/70 strains to macrolides (Table 2).

Furthermore, 82.85% (58/70) of *Streptoccocus pneumoniae* strains were characterized by multidrug resistance (non-susceptibility to at least one agent in three or more antimicrobial categories) (Table 3). A total of 64/70 strains showed resistance to trimethoprim/sulfamethoxazole, whereas only 3/70 strains were susceptible to all the tested antibiotics.

*Haemophilus influenzae*, the second most common bacteria found in the middle ear samples, accounted for 17 (17.52%) (Table 4). We noticed that all the strains were sensitive to a second-generation cephalosporin (cefuroxime) and to a third-generation cephalosporin (ceftriaxone). Using the nitrocefin test to assess beta-lactamase production, we identified only 4 (23.5%) ampicillin-resistant strains.

For the two strains of *Moraxella catarrhalis*, the nitrocefin test was positive. *Escherichia coli*, found in one sample, was sensitive to all antibiotics (ampicillin, amoxicillin/clavulanic acid, cefuroxime, ceftriaxone, gentamicin, amikacin, ciprofloxacin and trimethoprim/sulfamethoxazole). *Pseudomonas aeruginosa* (two samples) was also sensitive to all antibiotics (piperacillin/tazobactam, ceftazidime, imipenem, meropenem, gentamicin, ciprofloxacin).

The *Enterobacter aerogenes* strain, also found in only one sample, was a multidrug-resistant strain, sensitive only to piperacillin/tazobactam, carbapenems (imipenem, meropenem, ertapenem), amikacin and tetracycline, but resistant to a wide range of antibiotics: cephalosporins (cefuroxime, ceftazidime, ceftriaxone), aminoglycosides (gentamicin, tobramycin), fluoroquinolones (ciprofloxacin), ampicillin and amoxicillin/clavulanic acid. Out of 147 children enrolled in our study, 79 encountered treatment failure. *Streptococcus pneumoniae* was the most common microorganism involved in these cases (41 cases), followed by culture-negative cases (27 cases) and sporadic cases of other microorganisms (Table 5). Due to the lack of a national guideline regarding the treatment of AOM, the clinicians’ first choice in AOM treatment was characterized by a wide diversity: monotherapy (amoxicillin/clavulanic acid, ceftriaxone, cefuroxime or even clindamycin in cases of suspected mastoiditis) or associations of two antibiotics (ceftriaxone and ciprofloxacin, cefuroxime and clindamycin, cefuroxime and levofloxacin, clarithromycin and amoxicillin/clavulanic acid), indicated after 24 to 48 h of persistent symptomatology.

## 4. Discussions

Antimicrobial resistance still represents a global threat as there are more than 670,000 infections every year due to antibiotic-resistant microorganisms [13]. The list of global priority pathogens (GPP) published by the World Health Organization (WHO) includes, on the medium priority group, two of the major pathogens responsible for AOM in children: *Streptococcus pneumoniae* and *Haemophilus influenzae*, due to penicillin non-susceptibility and ampicillin-resistant strains, respectively [14].

After the incorporation of pneumococcal conjugate vaccines into national immunization programs worldwide, a significant reduction in AOM episodes has been recorded. In the United States of America, after the introduction of pneumococcal conjugate vaccine 7 (PCV7), the frequency of otitis media has reduced by 28% and the antimicrobial prescriptions decreased by 42% [15]. Similarly, in the United Kingdom, Dagan et al. reported a 22% reduction in the incidence of otitis media in children younger than 10 years old following the introduction of PCV7, with an additional 19% reduction after PCV7 was replaced by PCV13 [16]. In Sweden, the incidence of severe otitis media in children aged less than 2 years old decreased by 32% after the introduction of PCV13 [17].

Besides these encouraging data in the frequency of otitis media strains, important decreases in antimicrobial-resistant vaccine-type pneumococcal infections have been reported: Jansen et al. investigated the impact of human vaccines on bacterial antimicrobial resistance, highlighting the importance of vaccines targeting *Streptococcus pneumoniae* for reducing antibiotic consumption and antibiotic-resistant bacterial strains [18]. In the United States of America, antibiotic non-susceptible invasive pneumococcal strains decreased between 2009 (immediately before PCV13 was introduced) and 2013, from 6.7 to 2.2 per 100,000 for all ages, and from 6.5 to 0.5 per 100,000 in children younger than 5 years [19].

According to the World Health Organization, the prevalence of nasopharyngeal carriage of *Streptococcus pneumoniae* in infants and young children ranges from 27% to 85%, with higher rates among children in low- and middle-income countries [20]. A study of pediatric nasopharyngeal pneumococcal carriage rates in Romania found an incidence of 25%, with 59% of the serotypes isolated being covered by PCV13. Moreover, according to the most recent European Antimicrobial Resistance Surveillance Network data, the national incidence of penicillin non-susceptible pneumococci in Romania increased from 27.9% in 2017 to 40% in 2018 [21]. Amoxicillin and amoxicillin–clavulanate represent the preferred therapeutic agents for AOM caused by *Streptococcus pneumoniae*, whereas cephalosporins (cefixime) are preferred in AOM caused by *Haemophilus influenzae* or *Moraxella catarrhalis* [22]. Although the clinical practice guidelines provide a standardized and systematic approach on AOM management, the (locally) resistant strains, associated with treatment failure or severe cases of AOM, may require specific recommendations. When drafting a local guideline, it would be useful to consider the regional antimicrobial resistance patterns, since most of the otitis in children do not undergo routine tympanocentesis. In order to promote antibiotic stewardship, the WHO underlines the importance of guidelines with recommendations based on a regional antibiotic resistance profile. Many guidelines promote antibiotic stewardship, but only some (the United States of America, Australia, Belgium, Finland, Italy, Netherlands, Poland, Sweden, Portugal, Spain) of the guidelines take into consideration regional antimicrobial resistance patterns [23]. We have previously reviewed 20 AOM clinical practice guidelines on AOM management and noticed that the watchful waiting approach is preferred to immediate antibiotic treatment in 16 of the 20 guidelines, in order to decrease the antimicrobial resistance phenomenon. We noted that amoxicillin is the most recommended first-line treatment (16/20 guidelines). A high dose of 80 to 90 mg/kg/day of amoxicillin is encouraged in order to provide sufficient levels of antibiotic treatment in the middle ear fluid and to eradicate most of the *Streptococcus pneumoniae* strains. Only three guidelines (Denmark, Norway and Sweden) encourage the utilization of oral penicillin as a first-line choice [23].

Nowadays, there is no global consensus on which is the best treatment for children with persistent and significant AOM symptoms despite at least 48 h of antibiotic treatment. Besides clinical reexamination and consideration of tympanocentesis, most of the examined guidelines recommend a high dose of amoxicillin–clavulanate [23]. Some guidelines recommend extending the duration of amoxicillin therapy with at least 5 days (the WHO), increasing the amoxicillin dose to a maximum of 90 mg/kg/day (Australia, Germany), switching to a second generation of cephalosporin such as cefuroxime (Italy, Portugal), to a third generation cephalosporin such as ceftriaxone (Finland, France), or to trimethoprim/sulfamethoxazole (Norway). As the inefficiency of beta-lactamase inhibitors (in a standard dose) against penicillin-resistant *Streptococcus pneumoniae* strains was proven, being based on different mechanisms of resistance and limited spectrum of action, higher doses are needed in order to overcome the resistance phenomenon [23].

Despite the availability of the guidelines, the misuse of broad-spectrum antibiotics continued [24]. In clinical practice, using broad-spectrum antimicrobials has proven its efficiency only in terms of reducing the rate of treatment failure, but with the price of increasing adverse effects and a negative impact on quality of life [25]. Effective strategies of implementation are needed in order to reduce the threat of the worrying number of cases with an unjustified use of antibiotics [26].

Geographical differences regarding antimicrobial susceptibility patterns have been reported, emphasizing the role of regional surveillance studies in antibiotic prescriptions [4]. In Europe, there are limited data regarding antibiotic resistance in otitis media. However, in Greece, Stamboulidis et al. suggest that the non-susceptibility rates of *Streptococcus pneumoniae* to penicillin increased in the post-vaccination era [27]. In Bulgaria, Setchanova et al. report a 68.1% rate of penicillin-resistant strains [28], whereas in Spain, the reported percentage is only 6.5% [29]. These differences are based on variations of vaccination rates, adherence to guidelines and even laboratory testing methods.

Moreover, the last update on NICE (The National Institute for Health and Care Excellence) AOM management guideline (March 2022) underlines the importance of antibiotic stewardship and includes new recommendation on analgesic eardrops for children who do not need immediate antibiotics (AOM with no perforation or otorrhoea) in order to reduce the local inflammation and to decrease the antibiotic consumption [30].

Regarding the antimicrobial resistance of microorganisms found in middle ear samples from children with AOM, Hullegie et al., in a systematic review including 19 studies (10,560 children), reported that *Streptococcus pneumoniae*, *Haemophilus influenzae* and *Staphylococcus aureus* were the most prevalent microorganisms, with 76% of positive cultures [31]. The antimicrobial resistance reports were limited to *Streptococcus pneumoniae*, with a non-susceptibility rate (resistant or intermediate strains) ranging from 0% to 65.8%. These large variations are based on different vaccination rates, accessibility to medical services or geographical specific patterns (e.g., high AOM rates in indigenous Australians) [32]. In our sample, the highest frequency of resistance in isolated pneumococci was recorded for trimethoprim/sulfamethoxazole (64/70 strains), followed by macrolides (61/70 strains) and lincosamides (54/70 strains), while the lowest incidence was recorded for fluoroquinolones and vancomycin (0/70 strains). Various mechanisms of resistance have been discussed regarding these antibiotic classes: dihydropteroate synthase gene mutation, which is responsible for the occurrence of resistance to trimethoprim/sulfamethoxazole [33], and ribosomal demethylation or mutations of the ribosomal target site in macrolides [34]. The high proportion of the pneumococcal isolates’ resistance to trimethoprim/sulfamethoxazole (91.42%) is consistent with another retrospective study based on microbiological testing in children with AOM from our country: the highest non-susceptibility rate was also reported for trimethoprim/sulfamethoxazole (75%), but with lower non-susceptibility rates for macrolides (58.3% vs. 87.6% in our sample) and lincosamides (35.4% vs. 77.14% in our sample) [35]. Similarly, multidrug resistance of *Streptococcus pneumoniae* strains was detected in most cases (68.7% vs. 82.85% in our sample) [35].

However, based on the results of a meta-analysis of the studies on the antimicrobial resistance patterns in AOM in children, the recommendation of using amoxicillin as a first choice in treating children with AOM is still reasonable as the resistance of *Streptococcus pneumoniae* to penicillin is not usually associated to resistance to amoxicillin [36].

The culture-negative cases who required hospitalization due to positive criteria for acute otitis media (acute onset of the symptoms—fever, irritability, otalgia or otorrhea and middle ear effusion confirmed by otoscopy) raised particular awareness to us. It has been stated that conventional culture methods detect AOM pathogens in only 60% to 70% of cases [37]. Additionally, nowadays, the antimicrobial resistance phenomenon implies more evolved mechanisms, such as biofilms, which require special techniques for detection, such as scanning electron microscopy and fluorescent in situ hybridization, which are not routinely recommended [38].

## 5. Conclusions

The main bacteria involved in AOM etiology remain to be *Streptococcus pneumoniae*, *Haemophilus influenzae* and *Moraxella catarrhalis*. Our retrospective, tympanocentesis-based study revealed a worrying proportion of 82.85% (58/70) multidrug-resistant *Streptococcus pneumoniae* strains (non-susceptibility to at least one agent in three or more antimicrobial categories). The highest frequency of resistance in isolated pneumococci was recorded for trimethoprim/sulfamethoxazole (64/70 strains), followed by macrolides (61/70 strains) and lincosamides (54/70 strains), while the lowest incidence was recorded for fluoroquinolones and vancomycin (0/70 strains). Since global antibiotic consumption contributes to the emergence of antibiotic resistant bacteria, strategies to address the threats of antimicrobial resistance are needed to reduce the potential negative effects on hospitalization costs and duration.

The guidelines on AOM management reinforce the importance of antibiotics stewardship by encouraging no prescription or a delayed prescription of antibiotics. Recommendations based on geographically specific patterns of etiology may reduce the treatment failures, which are usually related to antibiotic resistant strains. The culture-negative scenarios but with persistent symptoms should be assessed by special techniques as microorganisms tend to find more and more sensitive mechanisms of survival.

## Figures and Tables

**Table 1:** Bacteriological flora isolated from the middle ear fluid of children with AOM.

Bacteria	Number of Isolated Bacteria (% of Positive Cultures)	Age Group (Average Age, Median Age) *	Male: Female Ratio **
*Streptococcus pneumoniae*	70 (72.16%)	2–48 (20, 20)	39:31 (1.25)
*Haemophilus influenzae*	17 (17.52%)	4–60 (30, 24)	12:5 (2.4)
*Moraxella catarrhalis*	2 (2.06%)	21–72 (46, 46)	2:0
MRSA	2 (2.06%)	2–13 (7, 7)	1:1
*Pseudomonas aeruginosa*	2 (2.06%)	14–24 (19, 19)	1:1
*Escherichia coli*	1 (1.03%)	12 (12, 12)	-
*Enterobacter aerogenes*	1 (1.03%)	6 (6, 6)	-
*Streptococcus pyogenes*	1 (1.03%)	72 (72, 72)	-
*Turicella otitidis*	1 (1.03%)	12 (12, 12)	-
Total strains	97 (100%)	2–84 (22, 19)	93:54 (1.72)

* The age is reported as months old. ** Not applicable in case of only one positive strain of that type.

**Table 2:** Antibiotic resistance of *Streptococcus pneumoniae* strains.

Antibiotic		MIC * (µg/mL)			Number of Strains		
		S (Sensitive)	I (Intermediate)	R (Resistant)	S	I	R
Penicillins	Penicillin	0.25	4	8	15	10	4
		1	-	-	27	-	-
		2	-	-	14	-	-
Cephalosporins	Ceftriaxone	0.5–1	-	-	70	-	-
	Cefotaxime	0.25–0.5	2	-	69	1	-
	Cefuroxime	0.25	2	4	65	1	4
Macrolides	Azithromycin	<0.12	-	>2	9	-	61
	Clarithromycin	<0.12	-	>2	9	-	61
	Erythromycin	≤0.06	-	>1	9	-	61
Lincosamides	Clindamycin	<0.06	-	>1	16	-	54
Fluoroquinolones	Moxifloxacin	<0.25–0.5	-	-	70	-	-
	Levofloxacin	<0.5	4	-	69	1	-
Glycopeptide	Vancomycin	0.5–1	-	-	70	-	-
Trimethoprim/sulfamethoxazole		<2.5/4.7	-	>2/38	6	-	64
Chloraphenicol		<2	-	>8	66	-	4

* MIC—minimum inhibitory concentration.

**Table 3:** Multidrug resistance patterns of *Streptoccocus pneumoniae* strains.

Resistance Pattern	Classes of Resistance	Number of Strains (*n* = 70)
MDR (*n* = 58)	TMP/SMX, macrolides, lincosamides	46
	Penicillin, TMP/SMX, macrolides	4
	TMP/SMX, macrolides, lincosamides, cephalosporins	4
	TMP/SMX, macrolides, lincosamides, cloraphenicol	4
non-MDR (*n* = 12)	Susceptible to all the tested antibiotics	3
	TMP/SMX	6
	Macrolides	3

MDR, multidrug resistance; non-MDR, non-multidrug resistance; TMP/SMX, trimethoprim/sulfamethoxazole.

**Table 4:** Antibiotic resistance of *Haemophilus influenzae* strains.

Antibiotic	MIC * (µg/mL)		Number of Strains	
	S (Sensitive)	R (Resistant)	S	R
Amoxicillin/clavulanic acid	≤2/1	>8/4	14	3
Cefuroxime	<4	-	17	-
Ceftriaxone	≤2	-	17	-
Trimethoprim/sulfamethoxazole	≤0.5/9.5	≥4/76	8	9

* MIC—minimum inhibitory concentration.

**Table 5:** Microbiological profile in treatment failure cases.

Bacteria	Strain Number	Age Group (Average Age, Median Age) *	Antibiotic Resistant Classes
*Streptococcus pneumoniae*	41	6–48 (31, 16)	TMP/SMX, macrolides, lincosamides (33 strains)
			penicillin, TMP/SMX, macrolides (4 strains)
			TMP/SMX, macrolides, lincosamides, cephalosporins (4 strains)
Culture negative	27	5–84 (20, 15)	not applicable
*Haemophilus influenzae*	7	7–48 (20, 13)	amoxicillin/clavulanic acid (3 strains)
			TMP/SMX (4 strains)
MRSA	1	2	methicillin-resistant strain
*Enterobacter aerogenes*	1	6	MDR (cephalosporins, aminoglycosides, fluoroquinolones, ampicillin and amoxicillin/clavulanic acid.)
*Moraxella catarrhalis*	1	72	beta-lactams (penicillins, cephalosporins)
*Turicella otitidis*	1	12	not applicable **

* The age is reported as months old. ** *Turicella otitidis* has not been tested with antibiotics.

## Data Availability

The data presented in this study are available on request from the corresponding author. The data are not publicly available due to ethical issues.
